# Toward an Understanding of the Environmental and Public Health Impacts of Unconventional Natural Gas Development: A Categorical Assessment of the Peer-Reviewed Scientific Literature, 2009-2015

**DOI:** 10.1371/journal.pone.0154164

**Published:** 2016-04-20

**Authors:** Jake Hays, Seth B. C. Shonkoff

**Affiliations:** 1 PSE Healthy Energy, New York, New York, United States of America; 2 Department of Healthcare Policy and Research, Weill Cornell Medicine, New York, New York, United States of America; 3 PSE Healthy Energy, Oakland, California, United States of America; 4 Department of Environmental Science, Policy and Management, University of California, Berkeley, California, United States of America; 5 Lawrence Berkeley National Laboratory, Berkeley, California, United States of America; Institute for Health & the Environment, UNITED STATES

## Abstract

The body of science evaluating the potential impacts of unconventional natural gas development (UNGD) has grown significantly in recent years, although many data gaps remain. Still, a broad empirical understanding of the impacts is beginning to emerge amidst a swell of research. The present categorical assessment provides an overview of the peer-reviewed scientific literature from 2009–2015 as it relates to the potential impacts of UNGD on public health, water quality, and air quality. We have categorized all available original research during this time period in an attempt to understand the weight and direction of the scientific literature. Our results indicate that at least 685 papers have been published in peer-reviewed scientific journals that are relevant to assessing the impacts of UNGD. 84% of public health studies contain findings that indicate public health hazards, elevated risks, or adverse health outcomes; 69% of water quality studies contain findings that indicate potential, positive association, or actual incidence of water contamination; and 87% of air quality studies contain findings that indicate elevated air pollutant emissions and/or atmospheric concentrations. This paper demonstrates that the weight of the findings in the scientific literature indicates hazards and elevated risks to human health as well as possible adverse health outcomes associated with UNGD. There are limitations to this type of assessment and it is only intended to provide a snapshot of the scientific knowledge based on the available literature. However, this work can be used to identify themes that lie in or across studies, to prioritize future research, and to provide an empirical foundation for policy decisions.

## Introduction

Shale and tight gas development (known to nontechnical stakeholders as “fracking” and referred to herein as unconventional natural gas development, UNGD) continues to be the focus of controversy. Amidst economic and geopolitical considerations, the potential environmental and public health impacts of UNGD have received substantial attention in policy, media, and public debates. Claims of ground water contamination and adverse health outcomes have been widely cited and disputed, but what does the science actually show?

**Fig 1:**
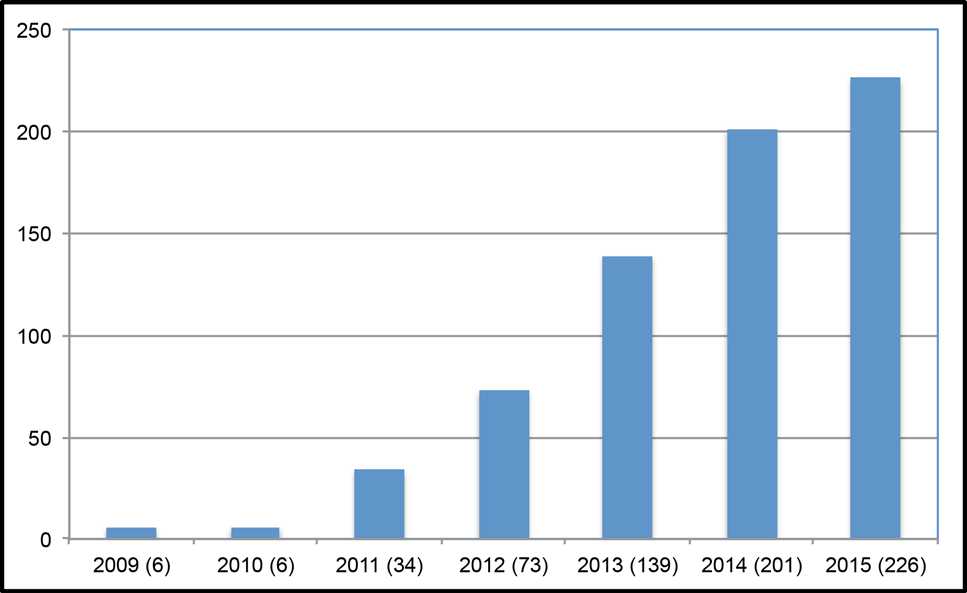
Number of publications that assess the impacts of UNGD per year, 2009–2015. At least 685 papers have been published in peer-reviewed scientific journals that are relevant to assessing the impacts of UNGD. The number of papers published per year has continually risen and at least 226 were published in 2015 alone.

While research continues to lag behind the rapid scaling of UNGD, there has been a surge of peer-reviewed scientific papers published in the past several years (Fig 1). By the end of 2015, over 80% of the peer reviewed scientific literature on shale and tight gas development has been published since January 1, 2013 and over 60% since January 1, 2014. This suggests an emerging understanding of the environmental and public health implications of UNGD in the scientific community. Yet, although numerous hazards and risks have been identified in studies to date, many data gaps remain. Notably, while there is now a far more substantive body of science than there was several years ago, there is still only a limited amount of epidemiology that explores associations between risk factors and health outcomes in human populations [1].

In this assessment we provide an overview, a current snapshot, of the scientific knowledge on potential environmental public health hazards, elevated risks, and outcomes associated with the development of shale and tight gas. We include only published, peer-reviewed literature available on the subject. More nuanced and systematic peer-reviewed public health review articles that provide greater levels of appraisal and analysis with in-depth narrative are available [2–4]. This particular assessment is intended to provide a broad understanding of the scientific literature in order to support the following goals:

- To understand the weight and direction of the scientific literature
- To provide comprehensive lists of studies in a field
- To identify themes that lie in or across individual studies
- To map and categorize existing literature for further review
- To prioritize future research and investigations

As activities continue to expand, counties, states, and nations are in a unique position to learn from experiences and scientific assessments conducted where UNGD is already underway [5,6]. While responsible energy policies require more than empirical data inputs [7,8], legislative and regulatory activities will benefit from the emerging body of science on the environmental and public health implications of UNGD. This assessment can be viewed as a summary of the peer-reviewed literature in order to help facilitate research efforts and inform policy discussions at the federal, state, and local levels.

## Methods

### Database assemblage and review

This assessment was conducted using the PSE Database on Shale and Tight Gas Development (available at: http://psehealthyenergy.org/site/view/1180 and referred to herein as the PSE Database). This near exhaustive collection of peer-reviewed scientific literature on the impacts of UNGD is divided into 12 topics: air quality, climate, community, ecology, economics, general, health, regulation, seismicity, waste/fluids, water quality, and water usage. We assembled this database over three years using a number of search strategies, including the following:

- Systematic searches in scientific databases across multiple disciplines:
  ○ PubMed (http://www.ncbi.nlm.nih.gov/pubmed/)
  ○ Web of Science (http://www.webofknowledge.com)
  ○ ScienceDirect (http://www.sciencedirect.com)
- Searches in existing collections of scientific literature on unconventional natural gas development, such as the Marcellus Shale Initiative Publications Database at Bucknell University (http://www.bucknell.edu/script/environmentalcenter/marcellus), complemented by Google (http://www.google.com) and Google Scholar (http://scholar.google.com)
- Manual searches (hand-searches) of references included in peer-reviewed studies and government reports that directly pertain to unconventional natural gas development.

For scientific literature search engines we used a combination of Medical Subject Headings (MeSH)-based and keyword strategies, which included the following terms as well as relevant combinations thereof:

shale gas, shale, hydraulic fracturing, fracking, drilling, natural gas, air pollution, methane, water pollution, health, public health, water contamination, fugitive emissions, air quality, climate, seismicity, waste, fluids, economics, ecology, water usage, regulation, community, epidemiology, Marcellus, Barnett, Fayetteville, Haynesville, Denver-Julesberg Basin, unconventional gas development, and environmental pathways.

Our database and this assessment excluded technical papers on UNGD not applicable to determining its potential impacts. Examples of literature that we excluded are engineering papers on optimal drilling strategies, petroleum reservoir evaluations, estimation algorithms of absorption capacity, patent efficacy assessments, and fracture models designed to inform stimulation techniques. Because our assessment is limited to papers subjected to external peer-review, it did not include government reports, environmental impact statements, policy briefs, white papers, law review articles, or other grey literature. Our assessment also excluded studies on some forms of UNGD, such as coalbed methane/coal seam gas as well as other forms of fossil fuel extraction that specifically exclude shale and/or tight gas development (e.g., tarsands, oil shale, etc.). While we are sure that we include the vast majority and certainly the most seminal studies on the environmental public health dimensions of UNGD in leading scientific journals, it is possible that a small number of publications are missing. As such, we refer to the literature database as *near* exhaustive.

The PSE Database has been used and reviewed by academics, experts, and government officials throughout the United States and internationally and has been subjected to public and professional scrutiny before and after this assessment. It represents the most comprehensive public collection of peer-reviewed scientific literature on shale and tight gas development available. Again, many of the publications in this database are discussed in greater detail in published review articles [2–4] and government reports [9,10].

### Scope of assessment

#### Definitions

There has been significant confusion about the environmental dimensions of UNGD (often termed “fracking”) because of the lack of uniform, well-defined terminology and boundaries of analysis [11]. The public and the media often use the term “fracking” as an umbrella term to refer to the entirety of UNGD (and often other forms of oil and gas development), including processes such as land clearing, well stimulation, hydrocarbon production, storage and transportation, and waste disposal. On the other hand, the oil and gas industry and many in the scientific community generally use the term as shorthand for one particular type of well stimulation method used to enhance the production of oil and natural gas: hydraulic fracturing.

The PSE Database and this assessment are focused on UNGD in its entirety, and not only the method of well stimulation. Environmental and public health assessments that include only the latter should have a limited role in policy discussions. In order to understand the environmental and public health dimensions of UNGD any reasonable approach must engage beyond a narrow view of only the well stimulation process of hydraulic fracturing, especially when the scientific literature indicates that other UNGD processes warrant greater concern. As such, the boundaries of our assessment include scientific literature on hydraulic fracturing *and* the associated operations and ancillary infrastructure required to develop and distribute unconventional natural gas. Although we use the term UNGD to refer to shale and tight gas development, some of the studies included in this report may either include data from, or be applicable to, other forms of UNGD enabled by hydraulic fracturing. Again, those focused solely on coal seam gas are beyond the scope of this assessment.

#### Inclusion and exclusion criteria

The temporal focus of this assessment was between 1 January 2009 and 31 December 2015 in order to capture what we believe to be the entirety of the published peer reviewed science on environmental public health dimensions of UNGD for this time period. We did not include papers released in 2015 ahead of print that will be published in 2016. We included original studies that evaluate environmental and public health hazards, risks, and impacts of UNGD, narrowly defined as shale or tight gas development (Table 1).

**Table 1:** Inclusion and Exclusion Criteria.

	Included	Excluded
**Type of unconventional fossil fuel development**	shale gas, tight gas	coal bed methane (coal seam gas), tar sands (oil sands), shale oil, shale (tight) oil*
**Type of publication**	scientific, peer-reviewed, original research	review articles, commentaries, government reports, environmental impact statements, white papers, law review articles, and other grey literature
**Date of publication**	published between 2009 and 2015	published prior to 2009 or since January 1, 2016
**Type of original research**	re: hazards, risks, and/or impacts to public health, water quality, and air quality	re: hazards, risks, and/or impacts to climate, community, ecology, economics, regulation, seismicity, water usage; baseline data; research methodology; technical papers (optimal drilling strategies, estimation algorithms of absorption capacity, etc.)

* Some of the air quality studies in Western oil and gas fields included unconventional fossil fuel development types other than shale and tight gas.

The majority of publications in the PSE Database are not considered in this assessment and we excluded the following topics: climate, community, ecology, economics, regulation, seismicity, waste/fluids, and water usage. Although many of these topics also have public health implications (e.g., climate change, economics, water usage, etc.), we have focused this assessment on original research that directly pertains to 1) public health, 2) water quality, and 3) air quality. We excluded some studies that may be located in the three topics used in this assessment, such as those that only provide baseline data or address research methods but fail to assess hazards, risks, or associated impacts.

As previously mentioned, we restricted the studies included in this assessment to those published from 1 January 2009 through 31 December 2015. There are studies on conventional forms of oil and natural gas development that are relevant to the public health dimensions of UNGD, but to maintain greater consistency we excluded those prior to 2009 from the assessment. For example, we did not include a study published in *The Lancet* that examined the association between testicular cancer and employment in agriculture and oil and gas development published in 1984 [12].

Relatedly, the scope of some of the studies we included in this assessment may go beyond shale and tight gas and could potentially include other forms of both conventional and unconventional oil and gas development. For instance, some of the top-down, field-based air pollution studies that gauge leakage rates and emission factors in Western oil and gas fields [13,14]. We included studies not exclusively related to UNGD only when the focus of the studies is relevant and they were published within our specified timeframe. For instance, studies that measured VOC emissions in a region with shale gas development as well as other forms of conventional and unconventional oil and gas development were included in this assessment.

Lastly, we only included original research in our assessment. We considered research original if it measured potential or actual health outcomes or complaints and air quality and water quality assessments related to UNGD. We excluded literature that attempted to determine public opinion or that considered methods for future research agendas.

### Categorical framework

We have created binary categories for each topic in an attempt to identify and group studies in an intuitive way that focuses on the indication of what might be considered to be a relevant or significant impact. Some of the studies categorized belong in more than one topic. For instance, studies that contain data that are relevant to both air quality and public health are included in both of these topics [15–17].

As with any scientific analysis there is also a qualitative component in our operational definitions and methods of categorization (Table 2). It is possible that some may disagree as to what constitute findings that indicate a public health hazard or elevated risk. To address this concern we have listed specific criteria of what would qualify a study for inclusion in a particular category within each relevant section below. Examples include statistically significant positive associations between UNGD or a particular health outcome or measurements documented above recommended air quality standards. In some cases, the relative significance of an impact related to UNGD is based on the interpretation of the evidence by the authors of the study. Readers may also refer to the tables included in the appendix for citations and categorization of the studies, which are listed alphabetically by author in each topic (S1 Appendix).

**Table 2:** Categorical Framework.

Topics	Categories	
	A	B
**Public Health**	Findings that indicate public health hazards, elevated risks, or adverse health outcomes	Findings that indicate no significant public health hazards, elevated risks, or adverse health outcomes
**Water Quality**	Findings that indicate potential, positive association, or actual incidence of water contamination	Findings that indicate minimal potential, no association, or rare incidence of water contamination
**Air Quality**	Findings that indicate elevated air pollutant emissions and/or atmospheric concentrations	Findings that indicate no significantly elevated air pollutant emissions and/or atmospheric concentrations

Our approach often does not account for various nuances in the results of particular studies. For instance, some studies may contain findings of both positive associations and no associations between UNGD and particular health outcomes. In our assessment we chose to include a study with any positive finding or indication of a particular impact in Category A. As such, a study that found an association between UNGD and health endpoint X, but no association with health endpoints Y and Z, would still be included in Category A.

#### Public Health

Studies that assess public health risks and endpoints, including epidemiologic investigations, continue to be particularly limited compared to studies of public health hazards. To date, most of the peer-reviewed health oriented publications are commentaries and literature reviews. In this topic we included original research that considers the question of public health in the context of UNGD. Of course, empirical findings in other categories such as air quality and water quality are relevant to public health. However, in this topic we only include those studies that directly consider the health of human populations and individuals as well as studies that examine animal health as they can provide sentinel information for human health risks.

In this topic we consider research “original” if it measures potential or actual health outcomes or complaints (i.e., not health research that only attempts to determine public opinion or consider methods for future research agendas). In addition to epidemiology, we included studies in this topic that focus primarily on environmental monitoring, but which also contain significant discussion about public health risks or outcomes [15,18,19]. In some of these cases, we have cross-listed the study within the water or air quality topic.

For the public health topic, we placed a study in category A or B based on whether or not it provided evidence, documentation, or acknowledgment of any of the following that are attributed to UNGD:

- A positive association with at least one adverse health outcome (e.g., birth defects, hospitalization)
- A positive association with a known human health risk (e.g., elevated benzene concentrations)
- Increased health risks from exposure to pollutant emissions
- A positive association with reported health symptoms in randomized survey proximity analysis
- Self-reported health symptoms or complaints in humans or animals;
- Toxicological concerns in the context of protective limitations (e.g, monitoring impediments)
- Explicit health concerns based on documented environmental contamination (e.g., endocrine disruption chemicals, high PAH levels in ambient air, etc.)

#### Water Quality

The allocation of water quality studies to binary categories is more complex than those focused on human health in that some rely on empirical field measurements, while others explore mechanisms for contamination or use modeled data to assess or predict water quality risks. Some of these studies explored only one aspect of UNGD, such as waste disposal or the well stimulation process enabled by hydraulic fracturing. These studies did not always indicate whether or not UNGD as a whole is associated with water contamination and are therefore limited in their utility for gauging water quality impacts. Nonetheless, we included all original research, including modeling studies as well as those that consider contamination mechanisms and/or exposure pathways. We excluded studies that explored only evaluative methodology or baseline assessments prior to UNGD as well as papers that only comment on or review previous studies. Here we were only concerned with actual findings in the field or modeling studies that specifically address the risk or potential occurrence of water contamination.

For this topic, we placed a study in category A or B based on whether or not it provided evidence, documentation, or acknowledgment of any of the following that are attributed to UNGD:

- A positive association with water contamination (e.g., proximity analysis showing increased concentrations of methane, heavy metals, salinity, etc.)
- Elevated surface or groundwater pollutant concentrations resulting from fluid releases or wastewater treatment/disposal
- Plausible contamination pathways and potential for water quality impacts from risk assessment/analysis of failure mechanism (e.g., casing and cement impairment)
- Plausible contamination pathways and potential for water quality impacts from modeling or geochemical evidence
- Water quality impacts based on analysis of microbial communities
- A significant quantity of reported incidents of water contamination relative to development activity

#### Air Quality

The papers included in the air quality assessment are those that specifically address air pollutant emissions and atmospheric concentrations from UNGD at either a local or regional scales. These papers primarily include measurements of local and regional emissions and atmospheric concentrations of non-methane volatile organic compounds, hazardous air pollutants, and tropospheric ozone attributable to upstream natural gas, and sometimes oil, activities since atmospheric measurements usually account for both.

Although methane is a precursor to global background tropospheric ozone concentrations we excluded studies that focus exclusively on methane emissions from this topic. We do, however, include studies that measure emissions of methane *and* non-methane volatile organic compounds (VOC), given the known health-damaging dimensions of a number of VOCs (i.e., benzene, toluene, ethylbenzene, xylene, 1,3 butadiene, acetaldehyde, etc.) and the role of light alkane VOCs in the production of the respiratory irritant, tropospheric (ground-level) ozone. We included a few studies that explore the public health risks associated with air pollutant emissions in both the air and the public health categories.

For this topic, we placed a study in category A or B based on whether or not it provided evidence, documentation, or acknowledgment of any of the following that are attributed to UNGD:

- Measurement(s) or estimation(s) of emissions or atmospheric concentration in excess of recommended air quality standards (e.g., NAAQS, federal ozone standards, etc.)
- Emission estimates that are significantly elevated above state emission inventory estimates
- Public health risks due to toxic air emissions or ambient air concentrations
- Measurement of emissions and/or atmospheric concentrations highly elevated over regional background

## Results

### Public Health

**Fig 2:**
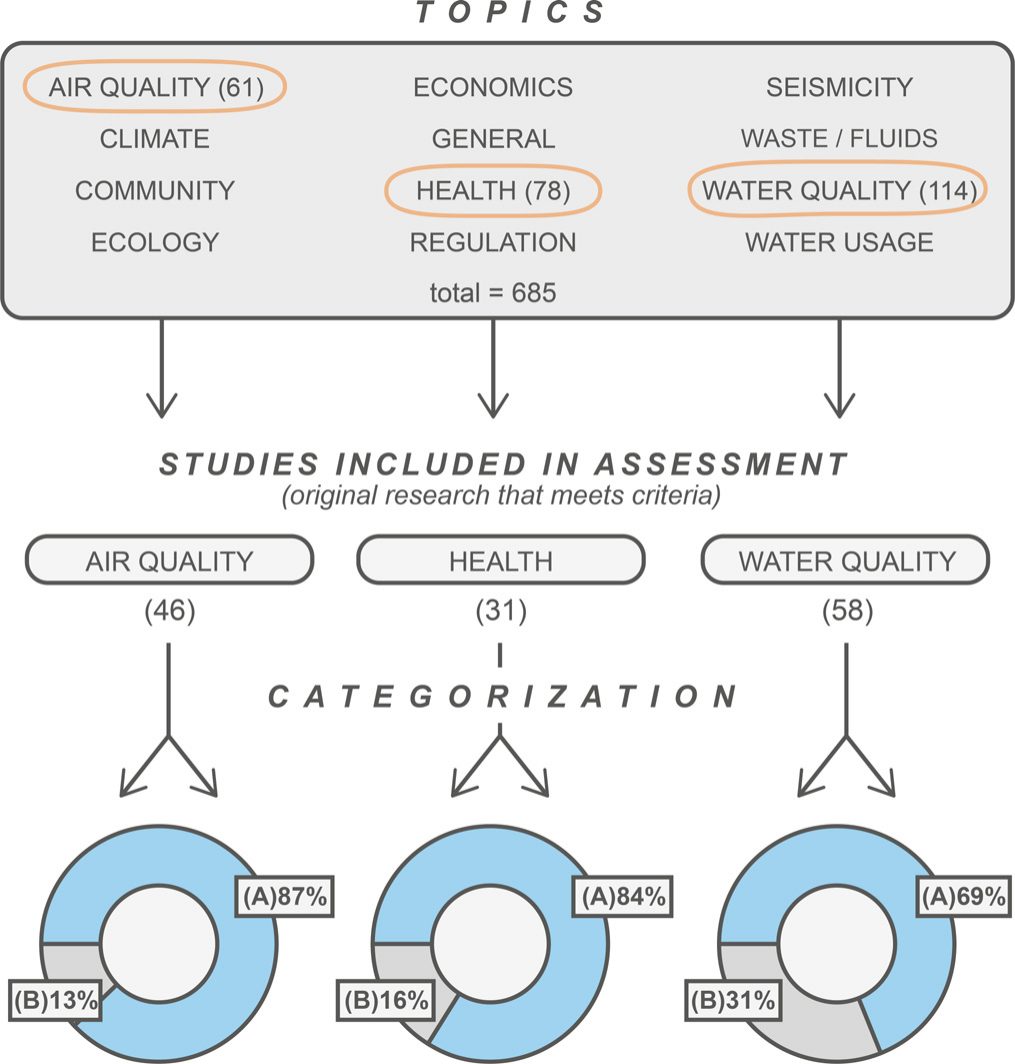
Selection Process and Results. This assessment draws from the peer-reviewed literature for three topics in the PSE Database: Air Quality, Health, and Water Quality. Of the 61 publications in air quality, 46 met our criteria; of the 78 publications in health, 31 met our criteria; and of the 114 publications in water quality, 58 met our criteria. From here we placed the original research that met our criteria into one of two categories (see Table 2). Our results indicate that 84% of public health studies contain findings that that indicate public health hazards, elevated risks, or adverse health outcomes, 69% of water quality studies contain findings that indicate potential, positive association, or actual incidence of water contamination, and 87% of air quality studies contain findings that indicate elevated air pollutant emissions and/or atmospheric concentrations.

Based on our criteria, we included 31 original research studies relevant to UNGD and public health hazards, risks, and health outcomes. Of these 31 studies, 26 (84%) contain findings that indicate public health hazards, elevated risks, or adverse public health outcomes and 5 (16%) contain findings that indicate no significant public health hazards, elevated risks, or adverse health outcomes associated with UNGD (Fig 2). The vast majority of all papers on this topic indicate the need for additional study, particularly large-scale, quantitative epidemiologic research.

### Water Quality

Based on our criteria, we included 58 original research studies relevant to shale gas development and water quality. Of these 58 studies, 40 (69%) have findings that indicate potential, positive association, or actual incidence of water contamination associated with UNGD, while 18 (31%) have findings that indicate minimal potential, no association, or rare incidence of water contamination (Fig 2).

### Air Quality

Based on our criteria, we included 46 original research studies relevant to questions involving associations between UNGD and air pollutant emissions and atmospheric air pollutant concentrations. Of these 46 studies, 40 (87%) have findings that indicate that UNGD increased air pollutant emissions and/or atmospheric concentrations, while 6 (12%) of the studies contain findings that provide no indication of significantly elevated air pollutant emissions and/or atmospheric concentrations (Fig 2).

## Discussion

In this assessment, we reviewed the findings of original peer-reviewed research that evaluates associations between UNGD and air quality, water quality, and public health to determine the direction of the scientific literature. For each topic we found that the majority of original research indicate hazards, elevated risks, or potential impacts from UNGD on the outcome of interest. These results suggest that UNGD may contribute to an environmental public health burden, which is consistent with numerous scientific review articles and government reports.

A review of the research included in this assessment can help identify themes that emerge in study design, methodology, hypotheses, scope, findings, and recommendations. With regard to the latter, one one theme that continually emerged was a recommendation for additional empirical investigations to better understand the risks to water, air, and public health presented by UNGD. Other themes included the recognized need among researchers for baseline studies to allow for before and after comparative assessments and longitudinal data to determine potential short- and long-term impacts.

Numerous data gaps on the environmental and public health impacts of UNGD exist, many of which have already been recognized in the scientific literature. Several notable data gaps are worth mentioning, however, and the following remain largely unknown: the extent to which the presence of stray-gas in aquifers indicates the potential for chemical contamination from hydraulic fracturing fluids; changes in well integrity failure rates over time; the legacy effects and relative contribution of air pollutants emissions from aging and abandoned wells; exposure data to characterize the frequency, duration, and degree of exposure to various stressors; community health risks from physical hazards (e.g., light and noise); and the overall magnitude of human-health risks.

The need for quantitative epidemiological research on this subject is widely recognized in the scientific community, but it is difficult to conduct until exposure parameters are better determined and reported cases of health outcomes are analyzed. Many epidemiological studies are expensive, time consuming, and often rely on data that are difficult to obtain. The fact that potential exposures would have taken place before background data could be collected only complicates the issue. Although there is quite a bit of evidence of hazards and elevated risks, drawing conclusions about the magnitude of health burdens attributable to UNGD remains difficult from an epidemiological perspective.

### Limitations

There are limitations to this assessment that relate to both its methods and the interpretation of its findings. As previously mentioned, the type of binary categorization we used may not account for the nuances of findings in many of these studies. Relatedly, this type of categorization effectively ranks the quality of the studies included in this article equally, despite clear differences in the weight and merit that should be ascribed to each study, based on either its design or interpretation of the evidence. Our work, however, was not intended to provide commentary on the quality of each study since here we are primarily concerned with the overall weight of the evidence. The quality and subsequent weight that should be given to a particular study are influenced by a number of factors, such as its design, methodology, and execution. We have only broadly surveyed original research across three different topics, including, but not limited to, qualitative epidemiology, risk analysis, in situ measurements, and modeling studies. There are strengths and weaknesses with each empirical method and it was not our aim to consider these attributes on an individual basis. Ultimately, this assessment relied on the peer-reviewed process itself in its consideration of the quality of the work. While not all peer-reviewed studies are of equal merit, this appeared to be the most simple, useful, and appropriate standard for quality control and consideration given our purposes.

Our selection criteria influence the categorization process and certain data inputs are gained or lost by our decisions to include or exclude particular type of studies. By only including original research on air quality, water quality, and public health, we are not accounting for all of the studies that may be pertinent to each topic (e.g., the existence or absence of elevated public health hazards, etc.). For instance, climate change, water usage, and economic gains may all influence environmental and public health outcomes. We have excluded these topics from our analysis and have chosen to focus only on the three that have consistently received the most attention among environmental public health researchers. Additionally, by not including government reports that do not appear in peer-reviewed journals we may be missing useful data and analysis that can inform UNGD public health implications as well as air and water quality concerns.

The majority of studies included in this assessment were conducted to determine whether or not adverse effects from UNGD exist. These types of investigations may, by their very nature, produce reporting or design bias. This is an inherent limitation of the scientific discipline; scientists are not immune from value judgments that shape research and scientific reasoning, including hypotheses to be tested, boundaries of analysis, and interpretation of evidence. Biases are difficult to account for in this context and we have chosen to rely on the peer-review process in this determination.

Furthermore, while the PSE Database is–to our best knowledge–exhaustive, our literature search may not have captured every relevant peer-reviewed scientific paper. Some journal articles are not always available in electronic databases or may be captured at a later time. As UNGD continues to gain the attention of the scientific community in other parts of the world, more and more research on the subject has been published in relatively obscure journals that may not be readily available. While we are confident that our MeSH-terms account for nearly all of the research on this topic, there is a possibility that some studies that use different or less traditional terminology may have been missed. We did our best to account for what may not have been initially discovered in an online database with manual searches of the scientific literature over a several year period.

Differences in geography, geology, petroleum reservoir type, and regulatory regime may also render some studies less relevant when interpreted across geographic space. Our assessment is only concerned with current empirical evidence in the peer-reviewed literature and we do not consider different regulatory regimes that could potentially influence environmental and public health outcomes in positive or negative ways. For instance, technological improvements such as universal deployment of reduced emission completions may mitigate some existing air pollutant emission issues.

Despite its limitations, our assessment provides a general understanding of the weight of the scientific evidence of possible impacts arising from UNGD that are relevant to environmental public health. It demonstrates that the weight of the scientific literature indicates that there are hazards and elevated risks to human health as well as possible adverse health outcomes.

Finally, it must be understood that all forms of energy production and industrial processing have environmental impacts. Our assessment is only focused on assessing the available science on the environmental and public health dimensions of the development of natural gas from shale and tight formations. We make no claims about the level of impact that should be tolerated by society–these are ultimately value judgments that incorporate more than empirical findings.

## Supporting Information

S1 Appendix List of studies included and excluded in assessment by topic. (DOCX)
