# Supplementary material for: Toward an Understanding of the Environmental and Public Health Impacts of Unconventional Natural Gas Development: A Categorical Assessment of the Peer-Reviewed Scientific Literature, 2009-2015
Source: PLoS One. 2016 Apr 20;11(4):e0154164. doi: 10.1371/journal.pone.0154164 (PMC4838293; doi:10.1371/journal.pone.0154164)
Supplement: S1 Appendix — (DOCX) [file pone.0154164.s001.docx]

**Appendix**

**Topic: Health**

Total in database: 78

Total excluded in assessment: 47

Total included in assessment: 31

Total in Category A: 26 (84%)

Total in Category B: 5 (16%)

| **Category A: Findings that indicate public health hazards, elevated risks, or adverse health outcomes (26)** |
| --- |
| 1. Bamberger M, Oswald RE. 2012. Impacts of Gas Drilling on Human and Animal Health. NEW SOLUTIONS: A Journal of Environmental and Occupational Health Policy 22:51–77; doi:10.2190/NS.22.1.e. 2. Bamberger M, Oswald RE. 2015. Long-term impacts of unconventional drilling operations on human and animal health. Journal of Environmental Science and Health 50: 447–459. 3. Brown DR, Lewis C, Weinberger BI. 2015. Human exposure to unconventional natural gas development: A public health demonstration of periodic high exposure to chemical mixtures in ambient air. Journal of Environmental Science and Health, Part A 50: 460–472. 4. Brown D, Weinberger B, Lewis C, Bonaparte H. 2014. Understanding exposure from natural gas drilling puts current air standards to the test. Rev Environ Health 29:277–292; doi:10.1515/reveh-2014-0002. 5. Casey JA, Ogburn EL, Rasmussen SG, Irving JK, Pollak J, Locke PA, et al. 2015a. Predictors of Indoor Radon Concentrations in Pennsylvania, 1989–2013. Environmental Health Perspectives; doi:10.1289/ehp.1409014. 6. Casey JA, Savitz DA, Rasmussen SG, Ogburn EL, Pollak J, Mercer DG, et al. 2015b. Unconventional Natural Gas Development and Birth Outcomes in Pennsylvania, USA: Epidemiology 1; doi:10.1097/EDE.0000000000000387. 7. Colborn T, Kwiatkowski C, Schultz K, Bachran M. 2011. Natural Gas Operations from a Public Health Perspective. Human and Ecological Risk Assessment: An International Journal 17:1039–1056; doi:10.1080/10807039.2011.605662. 8. Colborn T, Schultz K, Herrick L, Kwiatkowski C. 2014. An Exploratory Study of Air Quality near Natural Gas Operations. Human and Ecological Risk Assessment: An International Journal 20:86–105; doi:10.1080/10807039.2012.749447. 9. Esswein EJ, Breitenstein M, Snawder J, Kiefer M, Sieber WK. 2013. Occupational exposures to respirable crystalline silica during hydraulic fracturing. J Occup Environ Hyg 10:347–356; doi:10.1080/15459624.2013.788352. 10. Esswein EJ, Snawder J, King B, Breitenstein M, Alexander-Scott M, Kiefer M. 2014. Evaluation of Some Potential Chemical Exposure Risks During Flowback Operations in Unconventional Oil and Gas Extraction: Preliminary Results. Journal of Occupational and Environmental Hygiene 11:D174–D184; doi:10.1080/15459624.2014.933960. 11. Ferrar KJ, Kriesky J, Christen CL, Marshall LP, Malone SL, Sharma RK, et al. 2013. Assessment and longitudinal analysis of health impacts and stressors perceived to result from unconventional shale gas development in the Marcellus Shale region. International Journal of Occupational and Environmental Health 19:104–112; doi:10.1179/2049396713Y.0000000024. 12. Graham J, Irving J, Tang X, Sellers S, Crisp J, Horwitz D, et al. 2015. Increased traffic accident rates associated with shale gas drilling in Pennsylvania. Accident Analysis & Prevention 74:203–209; doi:10.1016/j.aap.2014.11.003. 13. Jemielita T, Gerton GL, Neidell M, Chillrud S, Yan B, Stute M, et al. 2015. Unconventional Gas and Oil Drilling Is Associated with Increased Hospital Utilization Rates. PLoS ONE 10:e0131093; doi:10.1371/journal.pone.0131093. 14. Kassotis CD, Klemp KC, Vu DC, Lin C-H, Meng C-X, Besch-Williford CL, et al. 2015a. Endocrine-Disrupting Activity of Hydraulic Fracturing Chemicals and Adverse Health Outcomes After Prenatal Exposure in Male Mice. Endocrinology en.2015–1375; doi:10.1210/en.2015-1375. 15. Kassotis CD, Tillitt DE, Davis JW, Hormann AM, Nagel SC. 2014. Estrogen and Androgen Receptor Activities of Hydraulic Fracturing Chemicals and Surface and Ground Water in a Drilling-Dense Region. Endocrinology 155:897–907; doi:10.1210/en.2013-1697. 16. Macey GP, Breech R, Chernaik M, Cox C, Larson D, Thomas D, et al. 2014. Air concentrations of volatile compounds near oil and gas production: a community-based exploratory study. Environmental Health 13:82; doi:10.1186/1476-069X-13-82. 17. McKenzie LM, Guo R, Witter RZ, Savitz DA, Newman LS, Adgate JL. 2014. Birth Outcomes and Maternal Residential Proximity to Natural Gas Development in Rural Colorado. Environmental Health Perspectives 122; doi:10.1289/ehp.1306722. 18. McKenzie LM, Witter RZ, Newman LS, Adgate JL. 2012. Human health risk assessment of air emissions from development of unconventional natural gas resources. Sci. Total Environ. 424:79–87; doi:10.1016/j.scitotenv.2012.02.018. 19. Paulik LB, Donald CE, Smith BW, Tidwell LG, Hobbie KA, Kincl L, et al. 2015. Impact of natural gas extraction on PAH levels in ambient air. Environ. Sci. Technol.; doi:10.1021/es506095e. 20. Rabinowitz PM, Slizovskiy IB, Lamers V, Trufan SJ, Holford TR, Dziura JD, et al. 2015. Proximity to Natural Gas Wells and Reported Health Status: Results of a Household Survey in Washington County, Pennsylvania. Environmental Health Perspectives 123:21–26; doi:10.1289/ehp.1307732. 21. Saberi P, Propert KJ, Powers M, Emmett E, Green-McKenzie J. 2014. Field Survey of Health Perception and Complaints of Pennsylvania Residents in the Marcellus Shale Region. Int J Environ Res Public Health 11:6517–6527; doi:10.3390/ijerph110606517. 22. Slizovskiy, Ilya B., Conti LA, Trufan SJ, Reif JS, Lamers VT, Stowe MH, et al. 2015. Reported health conditions in animals residing near natural gas wells in southwestern Pennsylvania. Journal of Environmental Science and Health, Part A 50: 473–481. 23. Stacy SL, Brink LL, Larkin JC, Sadovsky Y, Goldstein BD, Pitt BR, et al. 2015. Perinatal Outcomes and Unconventional Natural Gas Operations in Southwest Pennsylvania. PLoS ONE 10:e0126425; doi:10.1371/journal.pone.0126425. 24. Steinzor N, Subra W, Sumi L. 2013. Investigating Links between Shale Gas Development and Health Impacts Through a Community Survey Project in Pennsylvania. NEW SOLUTIONS: A Journal of Environmental and Occupational Health Policy 23:55–83; doi:10.2190/NS.23.1.e. 25. Williams JF, Lundy JB, Chung KK, Chan RK, King BT, Renz EM, et al. 2014. Traumatic Injuries Incidental to Hydraulic Well Fracturing: A Case Series. Journal of Burn Care & Research 1; doi:10.1097/BCR.0000000000000219. 26. Yao Y, Chen T, Shen SS, Niu Y, DesMarais TL, Linn R, et al. 2015. Malignant human cell transformation of Marcellus Shale gas drilling flow back water. Toxicology and Applied Pharmacology 288:121–30; doi:10.1016/j.taap.2015.07.011. |
| **Category B: Findings that indicate no significant public health hazards, elevated risks, or adverse health outcomes (5)** |
| 1. Bloomdahl R, Abualfaraj N, Olson M, Gurian PL. 2014. Assessing worker exposure to inhaled volatile organic compounds from Marcellus Shale flowback pits. J. Nat. Gas Sci. Eng. 21:348–356; doi:10.1016/j.jngse.2014.08.018. 2. Bunch AG, Perry CS, Abraham L, Wikoff DS, Tachovsky JA, Hixon JG, et al. 2014. Evaluation of impact of shale gas operations in the Barnett Shale region on volatile organic compounds in air and potential human health risks. Science of The Total Environment 468–469:832–842; doi:10.1016/j.scitotenv.2013.08.080. 3. Ethridge S, Bredfeldt T, Sheedy K, Shirley S, Lopez G, Honeycutt M. 2015. The Barnett Shale: From problem formulation to risk management. Journal of Unconventional Oil and Gas Resources; doi:10.1016/j.juogr.2015.06.001. 4. Fryzek J, Pastula S, Jiang X, Garabrant DH. 2013. Childhood cancer incidence in pennsylvania counties in relation to living in counties with hydraulic fracturing sites. J. Occup. Environ. Med. 55:796–801; doi:10.1097/JOM.0b013e318289ee02. 5. Zhang T, Hammack RW, Vidic RD. 2015. Fate of Radium in Marcellus Shale flowback water impoundments and assessment of associated health risks. Environ. Sci. Technol.; doi:10.1021/acs.est.5b01393. |
| **Excluded (47)** |
| 1. Adgate JL, Goldstein BD, McKenzie LM. 2014. Potential Public Health Hazards, Exposures and Health Effects from Unconventional Natural Gas Development. Environ. Sci. Technol. 48:8307–8320; doi:10.1021/es404621d. 2. Bamberger M, Oswald RE. 2014. Unconventional oil and gas extraction and animal health. Environ. Sci.: Processes Impacts; doi:10.1039/C4EM00150H. 3. Bolden AL, Kwiatkowski CF, Colborn T. 2015. New Look at BTEX: Are Ambient Levels a Problem? Environ. Sci. Technol. 49:5261–5276; doi:10.1021/es505316f. 4. Chalupka S. 2012. Occupational silica exposure in hydraulic fracturing. Workplace Health Saf 60:460; doi:10.3928/21650799-20120926-70. 5. Coram A, Moss J, Blashki G. 2014. Harms unknown: health uncertainties cast doubt on the role of unconventional gas in Australia’s energy future. Med. J. Aust. 200. 6. Down A, Armes M, Jackson RB. 2013. Shale Gas Extraction in North Carolina: Research Recommendations and Public Health Implications. Environ Health Perspect 121:A292–A293; doi:10.1289/ehp.1307402. 7. Fedak K, Gross S, Jacobsen M, Tvermoes B. 2014. Birth Outcomes and Natural Gas Development: Methodological Limitations. Environmental Health Perspectives 122:A232–A232; doi:10.1289/ehp.1408647. 8. Finkel M, Hays J, Law A. 2013a. The Shale Gas Boom and the Need for Rational Policy. American Journal of Public Health e1–e3; doi:10.2105/AJPH.2013.301285. 9. Finkel ML, Hays J. 2015. Environmental and health impacts of “fracking”: why epidemiological studies are necessary. J Epidemiol Community Health jech–2015–205487; doi:10.1136/jech-2015-205487. 10. Finkel ML, Hays J. 2013. The implications of unconventional drilling for natural gas: a global public health concern. Public Health 127:889–893; doi:10.1016/j.puhe.2013.07.005. 11. Finkel ML, Hays J, Law A. 2013b. Modern Natural Gas Development and Harm to Health: The Need for Proactive Public Health Policies. ISRN Public Health; doi:http://dx.doi.org/10.1155/2013/408658. 12. Finkel ML, Hays J, Law A. 2015. Unconventional natural gas development and human health: thoughts from the United States. Med. J. Aust. 203. 13. Finkel ML, Law A. 2011. The rush to drill for natural gas: a public health cautionary tale. Am J Public Health 101:784–785; doi:10.2105/AJPH.2010.300089. 14. Goldstein BD. 2014. The importance of public health agency independence: marcellus shale gas drilling in pennsylvania. Am J Public Health 104:e13–15; doi:10.2105/AJPH.2013.301755. 15. Goldstein BD, Kriesky J, Pavliakova B. 2012. Missing from the Table: Role of the Environmental Public Health Community in Governmental Advisory Commissions Related to Marcellus Shale Drilling. Environ Health Perspect 120:483–486; doi:10.1289/ehp.1104594. 16. Kaktins NM. 2011. Drilling the Marcellus shale for natural gas: environmental health issues for nursing. Pa Nurse 66: 4–8; quiz 8–9. 17. Kassotis CD, Tillitt DE, Lin C-H, McElroy JA, Nagel SC. 2015b. Endocrine-Disrupting Chemicals and Oil and Natural Gas Operations: Potential Environmental Contamination and Recommendations to Assess Complex Environmental Mixtures. Environmental Health Perspectives; doi:10.1289/ehp.1409535. 18. Korfmacher KS, Elam S, Gray KM, Haynes E, Hughes MH. 2014. Unconventional natural gas development and public health: toward a community-informed research agenda. Reviews on Environmental Health; doi:10.1515/reveh-2014-0049. 19. Korfmacher KS, Jones WA, Malone SL, Vinci LF. 2013. Public Health and High Volume Hydraulic Fracturing. NEW SOLUTIONS: A Journal of Environmental and Occupational Health Policy 23:13–31; doi:10.2190/NS.23.1.c. 20. Kovats S, Depledge M, Haines A, Fleming LE, Wilkinson P, Shonkoff SB, et al. 2014. The health implications of fracking. The Lancet 383:757–758; doi:10.1016/S0140-6736(13)62700-2. 21. Krzyzanowski J. 2012. Environmental pathways of potential impacts to human health from oil and gas development in northeast British Columbia, Canada. Environmental Reviews 20: 122–134. 22. Lauver LS. 2012. Environmental health advocacy: an overview of natural gas drilling in northeast Pennsylvania and implications for pediatric nursing. J Pediatr Nurs 27:383–389; doi:10.1016/j.pedn.2011.07.012. 23. Law A, Hays J, Shonkoff SB, Finkel ML. 2014. Public Health England’s draft report on shale gas extraction. BMJ 348:g2728–g2728; doi:10.1136/bmj.g2728. 24. Mackie P, Johnman C, Sim F. 2013. Hydraulic fracturing: a new public health problem 138 years in the making? Public Health 127:887–888; doi:10.1016/j.puhe.2013.09.009. 25. Mash R, Minnaar J, Mash B. 2014. Health and fracking: Should the medical profession be concerned? S. Afr. Med. J. 104: 332–335. 26. McCarron GP, King D. 2014. Unconventional natural gas development: economic salvation or looming public health disaster? Australian and New Zealand Journal of Public Health 38:108–109; doi:10.1111/1753-6405.12196. 27. McCawley M. 2015. Air Contaminants Associated with Potential Respiratory Effects from Unconventional Resource Development Activities. Semin Respir Crit Care Med 36:379–387; doi:10.1055/s-0035-1549453. 28. McDermott-Levy BR, Kaktins N, Sattler B. 2013. Fracking, the Environment, and Health. AJN, American Journal of Nursing 113:45–51; doi:10.1097/01.NAJ.0000431272.83277.f4. 29. McDermott-Levy R, Kaktins N. 2012. Preserving health in the Marcellus region. Pa Nurse 67: 4–10; quiz 11–12. 30. Meng Q. 2015. Spatial analysis of environment and population at risk of natural gas fracking in the state of Pennsylvania, USA. Science of The Total Environment 515–516:198–206; doi:10.1016/j.scitotenv.2015.02.030. 31. Mrdjen I, Lee J. 2015. High volume hydraulic fracturing operations: potential impacts on surface water and human health. Int J Environ Health Res 1–23; doi:10.1080/09603123.2015.1111314. 32. Ogneva-Himmelberger Y, Huang L. 2015. Spatial distribution of unconventional gas wells and human populations in the Marcellus Shale in the United States: Vulnerability analysis. Applied Geography 60:165–174; doi:10.1016/j.apgeog.2015.03.011. 33. Olaguer EP, Erickson M, Wijesinghe A, Neish B, Williams J, Colvin J. 2015. Updated Methods for Assessing the Impacts of Nearby Gas Drilling and Production on Neighborhood Air Quality and Human Health. J Air Waste Manag Assoc; doi:10.1080/10962247.2015.1083914. 34. Ovuakporaye S, Ojieh A, Ejebe D, Mordi J. 2012. Effect of Gas Flaring on Lung Function among Residents in Gas Flaring Community in Delta State, Nigeria. 35. Penning TM, Breysse PN, Gray K, Howarth M, Yan B. 2014. Environmental Health Research Recommendations from the Inter-Environmental Health Sciences Core Center Working Group on Unconventional Natural Gas Drilling Operations. Environmental Health Perspectives; doi:10.1289/ehp.1408207. 36. Perry SL. 2013. Using Ethnography to Monitor the Community Health Implications of Onshore Unconventional Oil and Gas Developments: Examples from Pennsylvania’s Marcellus Shale. NEW SOLUTIONS: A Journal of Environmental and Occupational Health Policy 23:33–53; doi:10.2190/NS.23.1.d. 37. Powers M, Saberi P, Pepino R, Strupp E, Bugos E, Cannuscio CC. 2015. Popular Epidemiology and “Fracking”: Citizens’ Concerns Regarding the Economic, Environmental, Health and Social Impacts of Unconventional Natural Gas Drilling Operations. J. Community Health 40:534–541; doi:10.1007/s10900-014-9968-x. 38. Rafferty MA, Limonik E. 2013. Is shale gas drilling an energy solution or public health crisis? Public Health Nurs 30:454–462; doi:10.1111/phn.12036. 39. Rosenman KD. 2014. Hydraulic Fracturing and the Risk of Silicosis: Clinical Pulmonary Medicine 21:167–172; doi:10.1097/CPM.0000000000000046. 40. Saberi P. 2013. Navigating Medical Issues in Shale Territory. NEW SOLUTIONS: A Journal of Environmental and Occupational Health Policy 23:209–221; doi:10.2190/NS.23.1.m. 41. Schmidt CW. 2011. Blind Rush? Shale Gas Boom Proceeds Amid Human Health Questions. Environ Health Perspect 119:a348–a353; doi:10.1289/ehp.119-a348. 42. Shonkoff SB, Hays J, Finkel ML. 2014. Environmental Public Health Dimensions of Shale and Tight Gas Development. Environmental Health Perspectives 122; doi:10.1289/ehp.1307866. 43. Walters K, Jacobson J, Kroening Z, Pierce C. 2015. PM2.5 Airborne Particulates Near Frac Sand Operations. J. Environ. Health 78: 8–12. 44. Webb E, Bushkin-Bedient S, Cheng A, Kassotis CD, Balise V, Nagel SC. 2014. Developmental and reproductive effects of chemicals associated with unconventional oil and natural gas operations. reveh 29:307–318; doi:10.1515/reveh-2014-0057. 45. Werner AK, Vink S, Watt K, Jagals P. 2015. Environmental health impacts of unconventional natural gas development: A review of the current strength of evidence. Science of The Total Environment 505:1127–1141; doi:10.1016/j.scitotenv.2014.10.084. 46. Witter RZ, McKenzie L, Stinson KE, Scott K, Newman LS, Adgate J. 2013. The use of health impact assessment for a community undergoing natural gas development. Am J Public Health 103:1002–1010; doi:10.2105/AJPH.2012.301017. 47. Witter RZ, Tenney L, Clark S, Newman LS. 2014. Occupational exposures in the oil and gas extraction industry: State of the science and research recommendations. Am. J. Ind. Med. n/a–n/a; doi:10.1002/ajim.22316. |

**Topic: Water Quality**

Total in database: 114

Total excluded in assessment: 56

Total included in assessment: 58

Total in Category A: 40 (69%)

Total in Category B: 18 (31%)

| **Category A: Findings that indicate potential, positive association, or actual incidence of water contamination (40)** |
| --- |
| 1. Alawattegama SK, Kondratyuk T, Krynock R, Bricker M, Rutter JK, Bain DJ, et al. 2015. Well water contamination in a rural community in southwestern Pennsylvania near unconventional shale gas extraction. Journal of Environmental Science and Health, Part A 50: 516–528. 2. Austin BJ, Hardgrave N, Inlander E, Gallipeau C, Entrekin S, Evans-White MA. 2015. Stream primary producers relate positively to watershed natural gas measures in north-central Arkansas streams. Science of The Total Environment 529:54–64; doi:10.1016/j.scitotenv.2015.05.030. 3. Bern CR, Clark ML, Schmidt TS, Holloway JM, McDougal RR. 2015. Soil disturbance as a driver of increased stream salinity in a semiarid watershed undergoing energy development. J. Hydrol. 524:123–136; doi:10.1016/j.jhydrol.2015.02.020. 4. Birdsell DT, Rajaram H, Dempsey D, Viswanathan HS. 2015. Hydraulic fracturing fluid migration in the subsurface: A review and expanded modeling results. Water Resour. Res. 51:7159–7188; doi:10.1002/2015WR017810. 5. Darrah TH, Vengosh A, Jackson RB, Warner NR, Poreda RJ. 2014. Noble gases identify the mechanisms of fugitive gas contamination in drinking-water wells overlying the Marcellus and Barnett Shales. PNAS 201322107; doi:10.1073/pnas.1322107111. 6. Davies RJ, Almond S, Ward RS, Jackson RB, Adams C, Worrall F, et al. 2014. Oil and gas wells and their integrity: Implications for shale and unconventional resource exploitation. Marine and Petroleum Geology 56:239–254; doi:10.1016/j.marpetgeo.2014.03.001. 7. Drollette BD, Hoelzer K, Warner NR, Darrah TH, Karatum O, O’Connor MP, et al. 2015. Elevated levels of diesel range organic compounds in groundwater near Marcellus gas operations are derived from surface activities. PNAS 201511474; doi:10.1073/pnas.1511474112. 8. Entrekin SA, Maloney KO, Kapo KE, Walters AW, Evans-White MA, Klemow KM. 2015. Stream Vulnerability to Widespread and Emergent Stressors: A Focus on Unconventional Oil and Gas. PLoS ONE 10:e0137416; doi:10.1371/journal.pone.0137416. 9. Ferrar KJ, Michanowicz DR, Christen CL, Mulcahy N, Malone SL, Sharma RK. 2013. Assessment of effluent contaminants from three facilities discharging Marcellus Shale wastewater to surface waters in Pennsylvania. Environ. Sci. Technol. 47:3472–3481; doi:10.1021/es301411q. 10. Fontenot BE, Hunt LR, Hildenbrand ZL, Carlton Jr. DD, Oka H, Walton JL, et al. 2013. An Evaluation of Water Quality in Private Drinking Water Wells Near Natural Gas Extraction Sites in the Barnett Shale Formation. Environ. Sci. Technol. 47:10032–10040; doi:10.1021/es4011724. 11. Gassiat C, Gleeson T, Lefebvre R, McKenzie J. 2013. Hydraulic fracturing in faulted sedimentary basins: Numerical simulation of potential contamination of shallow aquifers over long time scales. Water Resour. Res. 49:8310–8327; doi:10.1002/2013WR014287. 12. Grant CJ, Weimer AB, Marks NK, Perow ES, Oster JM, Brubaker KM, et al. 2015. Marcellus and mercury: Assessing potential impacts of unconventional natural gas extraction on aquatic ecosystems in northwestern Pennsylvania. Journal of Environmental Science and Health, Part A 50: 482–500. 13. Gross SA, Avens HJ, Banducci AM, Sahmel J, Panko JM, Tvermoes BE. 2013. Analysis of BTEX groundwater concentrations from surface spills associated with hydraulic fracturing operations. J Air Waste Manag Assoc 63: 424–432. 14. Heilweil VM, Grieve PL, Hynek SA, Brantley SL, Solomon DK, Risser DW. 2015. Stream Measurements Locate Thermogenic Methane Fluxes in Groundwater Discharge in an Area of Shale-Gas Development. Environ. Sci. Technol. 49:4057–4065; doi:10.1021/es503882b. 15. Heilweil VM, Stolp BJ, Kimball BA, Susong DD, Marston TM, Gardner PM. 2013. A Stream-Based Methane Monitoring Approach for Evaluating Groundwater Impacts Associated with Unconventional Gas Development. Groundwater 51:511–524; doi:10.1111/gwat.12079. 16. Hildenbrand ZL, Carlton DD, Fontenot B, Meik JM, Walton J, Taylor J, et al. 2015. A Comprehensive Analysis of Groundwater Quality in The Barnett Shale Region. Environ. Sci. Technol.; doi:10.1021/acs.est.5b01526. 17. Hladik ML, Focazio MJ, Engle M. 2014. Discharges of produced waters from oil and gas extraction via wastewater treatment plants are sources of disinfection by-products to receiving streams. Science of The Total Environment 466–467:1085–1093; doi:10.1016/j.scitotenv.2013.08.008. 18. Ingraffea AR, Wells MT, Santoro RL, Shonkoff SBC. 2014. Assessment and risk analysis of casing and cement impairment in oil and gas wells in Pennsylvania, 2000–2012. PNAS 201323422; doi:10.1073/pnas.1323422111. 19. Jackson RB, Vengosh A, Darrah TH, Warner NR, Down A, Poreda RJ, et al. 2013a. Increased stray gas abundance in a subset of drinking water wells near Marcellus shale gas extraction. PNAS 110:11250–11255; doi:10.1073/pnas.1221635110. 20. Johnson E, Austin BJ, Inlander E, Gallipeau C, Evans-White MA, Entrekin S. 2015a. Stream macroinvertebrate communities across a gradient of natural gas development in the Fayetteville Shale. Sci. Total Environ. 530-531C:323–332; doi:10.1016/j.scitotenv.2015.05.027. 21. Kang M, Baik E, Miller AR, Bandilla KW, Celia MK. 2015. Effective Permeabilities of Abandoned Oil and Gas Wells: Analysis of Data from Pennsylvania. Environ. Sci. Technol. 49:4757–4764; doi:10.1021/acs.est.5b00132. 22. Kassotis CD, Tillitt DE, Davis JW, Hormann AM, Nagel SC. 2014. Estrogen and Androgen Receptor Activities of Hydraulic Fracturing Chemicals and Surface and Ground Water in a Drilling-Dense Region. Endocrinology 155:897–907; doi:10.1210/en.2013-1697. 23. Llewellyn GT. 2014. Evidence and mechanisms for Appalachian Basin brine migration into shallow aquifers in NE Pennsylvania, USA. Hydrogeol J 22:1055–1066; doi:10.1007/s10040-014-1125-1. 24. Llewellyn GT, Dorman F, Westland JL, Yoxtheimer D, Grieve P, Sowers T, et al. 2015. Evaluating a groundwater supply contamination incident attributed to Marcellus Shale gas development. PNAS 201420279; doi:10.1073/pnas.1420279112. 25. Myers T. 2012. Potential Contaminant Pathways from Hydraulically Fractured Shale to Aquifers. Ground Water 50:872–882; doi:10.1111/j.1745-6584.2012.00933.x. 26. Olmstead SM, Muehlenbachs LA, Shih J-S, Chu Z, Krupnick AJ. 2013. Shale gas development impacts on surface water quality in Pennsylvania. Proc. Natl. Acad. Sci. U.S.A. 110:4962–4967; doi:10.1073/pnas.1213871110. 27. Osborn SG, Vengosh A, Warner NR, Jackson RB. 2011. Methane contamination of drinking water accompanying gas-well drilling and hydraulic fracturing. PNAS 108:8172–8176; doi:10.1073/pnas.1100682108. 28. Papoulias DM, Velasco AL. 2013. Histopathological Analysis of Fish from Acorn Fork Creek, Kentucky, Exposed to Hydraulic Fracturing Fluid Releases. Southeastern Naturalist 12:92–111; doi:10.1656/058.012.s413. 29. Parker KM, Zeng T, Harkness J, Vengosh A, Mitch WA. 2014. Enhanced Formation of Disinfection By-Products in Shale Gas Wastewater-Impacted Drinking Water Supplies. Environ. Sci. Technol.; doi:10.1021/es5028184. 30. Rahm BG, Vedachalam S, Bertoia LR, Mehta D, Vanka VS, Riha SJ. 2015. Shale gas operator violations in the Marcellus and what they tell us about water resource risks. Energy Policy 82:1–11; doi:10.1016/j.enpol.2015.02.033. 31. Reagan MT, Moridis GJ, Keen ND, Johnson JN. 2015. Numerical simulation of the environmental impact of hydraulic fracturing of tight/shale gas reservoirs on near-surface groundwater: Background, base cases, shallow reservoirs, short-term gas, and water transport. Water Resour. Res. 51:2543–2573; doi:10.1002/2014WR016086. 32. Rozell DJ, Reaven SJ. 2012. Water pollution risk associated with natural gas extraction from the Marcellus Shale. Risk Anal. 32:1382–1393; doi:10.1111/j.1539-6924.2011.01757.x. 33. Sang W, Stoof CR, Zhang W, Morales VL, Gao B, Kay RW, et al. 2014. Effect of Hydrofracking Fluid on Colloid Transport in the Unsaturated Zone. Environ. Sci. Technol.; doi:10.1021/es501441e. 34. Schwartz MO. 2014. Modelling the hypothetical methane-leakage in a shale-gas project and the impact on groundwater quality. Environ Earth Sci 73:4619–4632; doi:10.1007/s12665-014-3787-3. 35. Trexler R, Solomon C, Brislawn CJ, Wright JR, Rosenberger A, McClure EE, et al. 2014. Assessing impacts of unconventional natural gas extraction on microbial communities in headwater stream ecosystems in Northwestern Pennsylvania. Front. Microbiol 5:522; doi:10.3389/fmicb.2014.00522. 36. Warner NR, Christie CA, Jackson RB, Vengosh A. 2013a. Impacts of Shale Gas Wastewater Disposal on Water Quality in Western Pennsylvania. Environ. Sci. Technol.; doi:10.1021/es402165b. 37. Warner NR, Darrah TH, Jackson RB, Millot R, Kloppmann W, Vengosh A. 2014. New Tracers Identify Hydraulic Fracturing Fluids and Accidental Releases from Oil and Gas Operations. Environ. Sci. Technol.; doi:10.1021/es5032135. 38. Warner NR, Jackson RB, Darrah TH, Osborn SG, Down A, Zhao K, et al. 2012a. Geochemical evidence for possible natural migration of Marcellus Formation brine to shallow aquifers in Pennsylvania. PNAS; doi:10.1073/pnas.1121181109. 39. Zhang L, Anderson N, Dilmore R, Soeder DJ, Bromhal G. 2014. Leakage detection of Marcellus Shale natural gas at an Upper Devonian gas monitoring well: a 3-D numerical modeling approach. Environ. Sci. Technol.; doi:10.1021/es501997p. 40. Zhang L, Soeder DJ. 2015. Modeling of Methane Migration in Shallow Aquifers from Shale Gas Well Drilling. Ground Water; doi:10.1111/gwat.12361. |
| **Category B: Findings that indicate minimal potential, no association, or rare incidence of water contamination (18)** |
| 1. Bowen ZH, Oelsner GP, Cade BS, Gallegos TJ, Farag AM, Mott DN, et al. 2015. Assessment of surface water chloride and conductivity trends in areas of unconventional oil and gas development—Why existing national data sets cannot tell us what we would like to know. Water Resour. Res. 51:704–715; doi:10.1002/2014WR016382. 2. Brantley SL, Yoxtheimer D, Arjmand S, Grieve P, Vidic R, Pollak J, et al. 2014. Water resource impacts during unconventional shale gas development: The Pennsylvania experience. International Journal of Coal Geology; doi:10.1016/j.coal.2013.12.017. 3. Chabudzinski L, Chmiel S, Michalczyk Z. 2015. Metal content in the waters of the upper Sanna River catchment (SE Poland): condition associated with drilling of a shale gas exploration wellbore. Environ. Earth Sci. 74:6681–6691; doi:10.1007/s12665-015-4668-0. 4. Engelder T, Cathles LM, Bryndzia LT. 2014. The fate of residual treatment water in gas shale. Journal of Unconventional Oil and Gas Resources 7:33–48; doi:10.1016/j.juogr.2014.03.002. 5. Flewelling SA, Sharma M. 2014. Constraints on Upward Migration of Hydraulic Fracturing Fluid and Brine. Groundwater 52:9–19; doi:10.1111/gwat.12095. 6. Flewelling SA, Tymchak MP, Warpinski N. 2013. Hydraulic fracture height limits and fault interactions in tight oil and gas formations. Geophysical Research Letters 40:3602–3606; doi:10.1002/grl.50707. 7. Kohl CAK, Capo RC, Stewart BW, Wall AJ, Schroeder KT, Hammack RW, et al. 2014. Strontium Isotopes Test Long-Term Zonal Isolation of Injected and Marcellus Formation Water after Hydraulic Fracturing. Environ. Sci. Technol. 48:9867–9873; doi:10.1021/es501099k. 8. Li H, Carlson KH. 2014. Distribution and Origin of Groundwater Methane in the Wattenberg Oil and Gas Field of Northern Colorado. Environ. Sci. Technol. 48:1484–1491; doi:10.1021/es404668b. 9. Molofsky LJ, Connor JA, Wylie AS, Wagner T, Farhat SK. 2013. Evaluation of methane sources in groundwater in northeastern pennsylvania. Ground Water 51:333–349; doi:http://onlinelibrary.wiley.com/doi/10.1111/gwat.12056/abstract. 10. Nelson AW, Knight AW, Eitrheim ES, Schultz MK. 2015. Monitoring radionuclides in subsurface drinking water sources near unconventional drilling operations: a pilot study. Journal of Environmental Radioactivity 142:24–28; doi:10.1016/j.jenvrad.2015.01.004. 11. Nowamooz A, Lemieux J-M, Molson J, Therrien R. 2015. Numerical investigation of methane and formation fluid leakage along the casing of a decommissioned shale gas well. Water Resour. Res. 51:4592–4622; doi:10.1002/2014WR016146. 12. Pelak AJ, Sharma S. 2014. Surface water geochemical and isotopic variations in an area of accelerating Marcellus Shale gas development. Environmental Pollution 195:91–100; doi:10.1016/j.envpol.2014.08.016. 13. Reilly D, Singer D, Jefferson A, Eckstein Y. 2015. Identification of local groundwater pollution in northeastern Pennsylvania: Marcellus flowback or not? Environ. Earth Sci. 73: 8097–8109. 14. Sharma S, Bowman L, Schroeder K, Hammack R. 2014. Assessing changes in gas migration pathways at a hydraulic fracturing site: Example from Greene County, Pennsylvania, USA. Applied Geochemistry; doi:10.1016/j.apgeochem.2014.07.018. 15. Siegel DI, Azzolina NA, Smith BJ, Perry AE, Bothun RL. 2015a. Methane Concentrations in Water Wells Unrelated to Proximity to Existing Oil and Gas Wells in Northeastern Pennsylvania. Environ. Sci. Technol.; doi:10.1021/es505775c. 16. Skalak KJ, Engle MA, Rowan EL, Jolly GD, Conko KM, Benthem AJ, et al. 2014. Surface disposal of produced waters in western and southwestern Pennsylvania: Potential for accumulation of alkali-earth elements in sediments. International Journal of Coal Geology 126:162–170; doi:10.1016/j.coal.2013.12.001. 17. States S, Cyprych G, Stoner M, Wydra F, Kuchta J, Monnell J, et al. 2013. Brominated THMs in Drinking Water: A Possible Link to Marcellus Shale and Other Wastewaters. Journal - American Water Works Association 105:E432–E448; doi:10.5942/jawwa.2013.105.0093. 18. Warner NR, Kresse TM, Hays PD, Down A, Karr JD, Jackson RB, et al. 2013b. Geochemical and isotopic variations in shallow groundwater in areas of the Fayetteville Shale development, north-central Arkansas. Applied Geochemistry 35:207–220; doi:10.1016/j.apgeochem.2013.04.013. |
| **Excluded (56)** |
| 1. Amann-Hildenbrand A, Ghanizadeh A, Krooss BM. 2012. Transport properties of unconventional gas systems. Marine and Petroleum Geology 31:90–99; doi:10.1016/j.marpetgeo.2011.11.009. 2. Arent D, Logan J, Macknick J, Boyd W, Medlock KI, O’Sullivan F, et al. 2015. A review of water and greenhouse gas impacts of unconventional natural gas development in the United States. MRS Energy & Sustainability - A Review Journal 2; doi:10.1557/mre.2015.5. 3. Baldassare FJ, McCaffrey MA, Harper JA. 2014. A geochemical context for stray gas investigations in the northern Appalachian Basin: Implications of analyses of natural gases from Neogene-through Devonian-age strata. AAPG Bulletin 98:341–372; doi:10.1306/06111312178. 4. Brantley SL. 2015. Drinking water while fracking: now and in the future. Ground Water 53: 21–23. 5. Burton GA, Basu N, Ellis BR, Kapo KE, Entrekin S, Nadelhoffer K. 2014. Hydraulic “Fracking”: Are surface water impacts an ecological concern? Environ. Toxicol. Chem. 33:1679–1689; doi:10.1002/etc.2619. 6. Cohen HA, Parratt T, Andrews CB. 2013. Potential Contaminant Pathways from Hydraulically Fractured Shale to Aquifers. Groundwater 51:317–319; doi:10.1111/gwat.12015. 7. Darrah TH, Jackson RB, Vengosh A, Warner NR, Whyte CJ, Walsh TB, et al. 2015. The evolution of Devonian hydrocarbon gases in shallow aquifers of the northern Appalachian Basin: Insights from integrating noble gas and hydrocarbon geochemistry. Geochimica et Cosmochimica Acta 170:321–355; doi:10.1016/j.gca.2015.09.006. 8. Davies RJ, Mathias SA, Moss J, Hustoft S, Newport L. 2012. Hydraulic fractures: How far can they go? Marine and Petroleum Geology 37:1–6; doi:10.1016/j.marpetgeo.2012.04.001. 9. Down A, Schreglmann K, Plata DL, Elsner M, Warner NR, Vengosh A, et al. 2015. Pre-drilling background groundwater quality in the Deep River Triassic Basin of central North Carolina, USA. Applied Geochemistry 60:3–13; doi:10.1016/j.apgeochem.2015.01.018. 10. Engelder T. 2012. Capillary tension and imbibition sequester frack fluid in Marcellus gas shale. PNAS 109:E3625–E3625; doi:10.1073/pnas.1216133110. 11. Entrekin S, Evans-White M, Johnson B, Hagenbuch E. 2011. Rapid expansion of natural gas development poses a threat to surface waters. Frontiers in Ecology and the Environment 9:503–511; doi:10.1890/110053. 12. Ewers U, Gordalla B, Frimmel F. 2013. [Hydraulic fracturing - a hazard for drinking water?]. Gesundheitswesen 75:735–741; doi:10.1055/s-0033-1355369. 13. Farag AM, Harper DD. 2013. A review of environmental impacts of salts from produced waters on aquatic resources. International Journal of Coal Geology; doi:10.1016/j.coal.2013.12.006. 14. Gordalla BC, Ewers U, Frimmel FH. 2013. Hydraulic fracturing: a toxicological threat for groundwater and drinking-water? Environ Earth Sci 70:3875–3893; doi:10.1007/s12665-013-2672-9. 15. Gorody AW. 2012. Factors affecting the variability of stray gas concentration and composition in groundwater. Environmental Geosciences 19:17–31; doi:10.1306/eg.12081111013. 16. Hakala JA. 2014. Use of stable isotopes to identify sources of methane in Appalachian Basin shallow groundwaters: a review. Environ. Sci.: Processes Impacts; doi:10.1039/C4EM00140K. 17. Hamilton SM, Grasby SE, McIntosh JC, Osborn SG. 2015. The effect of long-term regional pumping on hydrochemistry and dissolved gas content in an undeveloped shale-gas-bearing aquifer in southwestern Ontario, Canada. Hydrogeol J 23:719–739; doi:10.1007/s10040-014-1229-7. 18. Hunt AG, Darrah TH, Poreda RJ. 2012. Determining the source and genetic fingerprint of natural gases using noble gas geochemistry: A northern Appalachian Basin case study. AAPG Bulletin 96:1785–1811; doi:10.1306/03161211093. 19. Jackson RB. 2014. The integrity of oil and gas wells. PNAS 201410786; doi:10.1073/pnas.1410786111. 20. Jackson RB, Lowry ER, Pickle A, Kang M, DiGiulio D, Zhao K. 2015. The Depths of Hydraulic Fracturing and Accompanying Water Use Across the United States. Environ. Sci. Technol.; doi:10.1021/acs.est.5b01228. 21. Jackson RE, Gorody AW, Mayer B, Roy JW, Ryan MC, Van Stempvoort DR. 2013b. Groundwater protection and unconventional gas extraction: the critical need for field-based hydrogeological research. Ground Water 51:488–510; doi:http://www.ncbi.nlm.nih.gov/pubmed/23745972. 22. Johnson JD, Graney JR, Capo RC, Stewart BW. 2015b. Identification and quantification of regional brine and road salt sources in watersheds along the New York/Pennsylvania border, USA. Applied Geochemistry 60:37–50; doi:10.1016/j.apgeochem.2014.08.002. 23. Kharak YK, Thordsen JJ, Conaway CH, Thomas RB. 2013. The Energy-Water Nexus: Potential Groundwater-Quality Degradation Associated with Production of Shale Gas. Procedia Earth and Planetary Science 7:417–422; doi:10.1016/j.proeps.2013.03.132. 24. Kissinger A, Helmig R, Ebigbo A, Class H, Lange T, Sauter M, et al. 2013. Hydraulic fracturing in unconventional gas reservoirs: risks in the geological system, part 2. Environ Earth Sci 70:3855–3873; doi:10.1007/s12665-013-2578-6. 25. Kretsinger Grabert V, Kaback DS, Briskin J, Brantley SL, Darrah TH, Jackson RB, et al. 2015. Unconventional Shale Gas Development and Potential Impacts to Groundwater. Groundwater 53:19–28; doi:10.1111/gwat.12307. 26. Krogulec E, Sawicka K. 2015. Groundwater protection in shale gas exploration areas - a Polish perspective. Episodes 38: 9–20. 27. Lange T, Sauter M, Heitfeld M, Schetelig K, Brosig K, Jahnke W, et al. 2013. Hydraulic fracturing in unconventional gas reservoirs: risks in the geological system part 1. Environ Earth Sci 70:3839–3853; doi:10.1007/s12665-013-2803-3. 28. Lautz LK, Hoke GD, Lu Z, Siegel DI, Christian K, Kessler JD, et al. 2014. Using Discriminant Analysis to Determine Sources of Salinity in Shallow Groundwater Prior to Hydraulic Fracturing. Environ. Sci. Technol.; doi:10.1021/es502244v. 29. Lavoie D, Rivard C, Lefebvre R, Séjourné S, Thériault R, Duchesne MJ, et al. 2013. The Utica Shale and gas play in southern Quebec: Geological and hydrogeological syntheses and methodological approaches to groundwater risk evaluation. International Journal of Coal Geology; doi:10.1016/j.coal.2013.10.011. 30. Liang C, Chen M, Jin Y, Lu Y. 2014. Wellbore stability model for shale gas reservoir considering the coupling of multi-weakness planes and porous flow. Journal of Natural Gas Science and Engineering 21:364–378; doi:10.1016/j.jngse.2014.08.025. 31. Loh LJ, Bandara GC, Weber GL, Remcho VT. 2015. Detection of water contamination from hydraulic fracturing wastewater: a μPAD for bromide analysis in natural waters. Analyst; doi:10.1039/C5AN00807G. 32. Long SC. 2014. Direct and indirect challenges for water quality from the hydraulic fracturing industry. J. Am. Water Work Assoc. 106:53–57; doi:10.5942/jawwa.2014.106.0155. 33. Lu Z, Hummel ST, Lautz LK, Hoke GD, Zhou X, Leone J, et al. 2015. Iodine as a sensitive tracer for detecting influence of organic-rich shale in shallow groundwater. Applied Geochemistry 60:29–36; doi:10.1016/j.apgeochem.2014.10.019. 34. Mauter MS, Alvarez PJJ, Burton GA, Cafaro DC, Chen W, Gregory KB, et al. 2014. Regional Variation in Water Related Impacts of Shale Gas Development and Implications for Emerging International Plays. Environ. Sci. Technol.; doi:10.1021/es405432k. 35. McMahon PB, Thomas JC, Hunt AG. 2013. Groundwater Ages and Mixing in the Piceance Basin Natural Gas Province, Colorado. Environ. Sci. Technol. 47:13250–13257; doi:10.1021/es402473c. 36. Moritz A, Helie J-F, Pinti D, Larocque M, Barnatche D, Retailleau S, et al. 2015. Methane baseline concentrations and sources in shallow aquifers from the shale gas-prone region of the St. Lawrence Lowlands (Quebec, Canada). Environ. Sci. Technol.; doi:10.1021/acs.est.5b00443. 37. Osborn SG, McIntosh JC. 2010. Chemical and isotopic tracers of the contribution of microbial gas in Devonian organic-rich shales and reservoir sandstones, northern Appalachian Basin. Applied Geochemistry 25:456–471; doi:10.1016/j.apgeochem.2010.01.001. 38. Pancras JP, Norris GA, Landis MS, Kovalcik KD, McGee JK, Kamal AS. 2015. Application of ICP-OES for evaluating energy extraction and production wastewater discharge impacts on surface waters in Western Pennsylvania. Science of The Total Environment 529:21–29; doi:10.1016/j.scitotenv.2015.04.011. 39. Penningroth SM, Yarrow MM, Figueroa AX, Bowen RJ, Delgado S. 2013. Community-Based Risk Assessment of Water Contamination from High-Volume Horizontal Hydraulic Fracturing. NEW SOLUTIONS: A Journal of Environmental and Occupational Health Policy 23:137–166; doi:10.2190/NS.23.1.i. 40. Rahm BG, Riha SJ. 2014. Evolving shale gas management: water resource risks, impacts, and lessons learned. Environ. Sci.: Processes Impacts; doi:10.1039/C4EM00018H. 41. Rahm BG, Riha SJ. 2012. Toward strategic management of shale gas development: Regional, collective impacts on water resources. Environmental Science & Policy 17:12–23; doi:10.1016/j.envsci.2011.12.004. 42. Ren L, Zhao J, Hu Y. 2014. Hydraulic Fracture Extending into Network in Shale: Reviewing Influence Factors and Their Mechanism. The Scientific World Journal 2014:e847107; doi:10.1155/2014/847107. 43. Révész KM, Breen KJ, Baldassare AJ, Burruss RC. 2010. Carbon and hydrogen isotopic evidence for the origin of combustible gases in water-supply wells in north-central Pennsylvania. Applied Geochemistry 25:1845–1859; doi:10.1016/j.apgeochem.2010.09.011. 44. Rhodes AL, Horton NJ. 2015. Establishing baseline water quality for household wells within the Marcellus Shale gas region, Susquehanna County, Pennsylvania, U.S.A. Applied Geochemistry; doi:10.1016/j.apgeochem.2015.03.004. 45. Roy J w., Ryan M c. 2013. Effects of Unconventional Gas Development on Groundwater: A Call for Total Dissolved Gas Pressure Field Measurements. Groundwater 51:480–482; doi:10.1111/gwat.12065. 46. Saba T, Orzechowski M. 2011. Lack of data to support a relationship between methane contamination of drinking water wells and hydraulic fracturing. PNAS 108:E663–E663; doi:10.1073/pnas.1108435108. 47. Saiers JE, Barth E. 2012. Potential Contaminant Pathways from Hydraulically Fractured Shale Aquifers. Ground Water 50:826–828; doi:10.1111/j.1745-6584.2012.00990.x. 48. Schon SC. 2011. Hydraulic fracturing not responsible for methane migration. PNAS 108:E664–E664; doi:10.1073/pnas.1107960108. 49. Sharma S, Mulder ML, Sack A, Schroeder K, Hammack R. 2013. Isotope Approach to Assess Hydrologic Connections During Marcellus Shale Drilling. Ground Water; doi:10.1111/gwat.12083. 50. Siegel DI, Smith B, Perry E, Bothun R, Hollingsworth M. 2015b. Pre-drilling water-quality data of groundwater prior to shale gas drilling in the Appalachian Basin: Analysis of the Chesapeake Energy Corporation dataset. Applied Geochemistry 63:37–57; doi:10.1016/j.apgeochem.2015.06.013. 51. Stephens DB. 2014. Analysis of the Groundwater Monitoring Controversy at the Pavillion, Wyoming Natural Gas Field. Ground Water; doi:10.1111/gwat.12272. 52. Vengosh A, Jackson RB, Warner N, Darrah TH, Kondash A. 2014. A Critical Review of the Risks to Water Resources from Unconventional Shale Gas Development and Hydraulic Fracturing in the United States. Environ. Sci. Technol.; doi:10.1021/es405118y. 53. Vengosh A, Warner N, Jackson R, Darrah T. 2013. The Effects of Shale Gas Exploration and Hydraulic Fracturing on the Quality of Water Resources in the United States. Procedia Earth and Planetary Science 7:863–866; doi:10.1016/j.proeps.2013.03.213. 54. Vidic RD, Brantley SL, Vandenbossche JM, Yoxtheimer D, Abad JD. 2013. Impact of Shale Gas Development on Regional Water Quality. Science 340; doi:10.1126/science.1235009. 55. Warner NR, Jackson RB, Darrah TH, Osborn SG, Down A, Zhao K, et al. 2012b. Reply to Engelder: Potential for fluid migration from the Marcellus Formation remains possible. PNAS 109:E3626–E3626; doi:10.1073/pnas.1217974110. 56. Wilson JM, Wang Y, VanBriesen JM. 2014. Sources of High Total Dissolved Solids to Drinking Water Supply in Southwestern Pennsylvania. J. Environ. Eng.-ASCE 140:B4014003; doi:10.1061/(ASCE)EE.1943-7870.0000733. |

**Topic: Air Quality**

Total in database: 61

Total excluded in assessment: 15

Total included in assessment: 46

Total in Category A: 40 (87%)

Total in Category B: 6 (13%)

| **Category A: Findings that indicate elevated air pollutant emissions and/or atmospheric concentrations (40)** |
| --- |
| 1. Ahmadi M, John K. 2015. Statistical evaluation of the impact of shale gas activities on ozone pollution in North Texas. Sci. Total Environ. 536:457–467; doi:10.1016/j.scitotenv.2015.06.114. 2. Ahmadov R, McKeen S, Trainer M, Banta R, Brewer A, Brown S, et al. 2015. Understanding high wintertime ozone pollution events in an oil- and natural gas-producing region of the western US. Atmos. Chem. Phys. 15:411–429; doi:10.5194/acp-15-411-2015. 3. Brantley HL, Thoma ED, Eisele AP. 2015. Assessment of volatile organic compound and hazardous air pollutant emissions from oil and natural gas well pads using mobile remote and on-site direct measurements. Journal of the Air & Waste Management Association 65:1072–1082; doi:10.1080/10962247.2015.1056888. 4. Brown DR, Lewis C, Weinberger BI. 2015. Human exposure to unconventional natural gas development: A public health demonstration of periodic high exposure to chemical mixtures in ambient air. Journal of Environmental Science and Health, Part A 50: 460–472. 5. Brown D, Weinberger B, Lewis C, Bonaparte H. 2014. Understanding exposure from natural gas drilling puts current air standards to the test. Rev Environ Health 29:277–292; doi:10.1515/reveh-2014-0002. 6. Carlton AG, Little E, Moeller M, Odoyo S, Shepson PB. 2014. The Data Gap: Can a Lack of Monitors Obscure Loss of Clean Air Act Benefits in Fracking Areas? Environ. Sci. Technol. 48:893–894; doi:10.1021/es405672t. 7. Eapi GR, Sabnis MS, Sattler ML. 2014. Mobile measurement of methane and hydrogen sulfide at natural gas production site fence lines in the Texas Barnett Shale. Journal of the Air & Waste Management Association 64:927–944; doi:10.1080/10962247.2014.907098. 8. Edwards PM, Brown SS, Roberts JM, Ahmadov R, Banta RM, deGouw JA, et al. 2014. High winter ozone pollution from carbonyl photolysis in an oil and gas basin. Nature 514:351–354; doi:10.1038/nature13767. 9. Edwards PM, Young CJ, Aikin K, deGouw JA, Dubé WP, Geiger F, et al. 2013. Ozone photochemistry in an oil and natural gas extraction region during winter: simulations of a snow-free season in the Uintah Basin, Utah. Atmospheric Chemistry and Physics Discussions 13:7503–7552; doi:10.5194/acpd-13-7503-2013. 10. Field RA, Soltis J, McCarthy MC, Murphy S, Montague DC. 2015. Influence of oil and gas field operations on spatial and temporal distributions of atmospheric non-methane hydrocarbons and their effect on ozone formation in winter. Atmos. Chem. Phys. 15:3527–3542; doi:10.5194/acp-15-3527-2015. 11. Franco B, Bader W, Toon GC, Bray C, Perrin A, Fischer EV, et al. 2015. Retrieval of ethane from ground-based FTIR solar spectra using improved spectroscopy: Recent burden increase above Jungfraujoch. Journal of Quantitative Spectroscopy and Radiative Transfer 160:36–49; doi:10.1016/j.jqsrt.2015.03.017. 12. Gilman JB, Lerner BM, Kuster WC, de Gouw JA. 2013. Source Signature of Volatile Organic Compounds from Oil and Natural Gas Operations in Northeastern Colorado. Environ. Sci. Technol. 47:1297–1305; doi:10.1021/es304119a. 13. Helmig D, Thompson CR, Evans J, Boylan P, Hueber J, Park J-H. 2014. Highly Elevated Atmospheric Levels of Volatile Organic Compounds in the Uintah Basin, Utah. Environ. Sci. Technol. 48:4707–4715; doi:10.1021/es405046r. 14. Kemball-Cook S, Bar-Ilan A, Grant J, Parker L, Jung J, Santamaria W, et al. 2010. Ozone Impacts of Natural Gas Development in the Haynesville Shale. Environ. Sci. Technol. 44:9357–9363; doi:10.1021/es1021137. 15. Koss AR, de Gouw J, Warneke C, Gilman JB, Lerner BM, Graus M, et al. 2015. Photochemical aging of volatile organic compounds associated with oil and natural gas extraction in the Uintah Basin, UT, during a wintertime ozone formation event. Atmos. Chem. Phys. 15:5727–5741; doi:10.5194/acp-15-5727-2015. 16. Lan X, Talbot R, Laine P, Torres A, Lefer B, Flynn J. 2015. Atmospheric Mercury in the Barnett Shale Area, Texas: Implications for Emissions from Oil and Gas Processing. Environ. Sci. Technol. 49:10692–10700; doi:10.1021/acs.est.5b02287. 17. Litovitz A, Curtright A, Abramzon S, Burger N, Samaras C. 2013. Estimation of regional air-quality damages from Marcellus Shale natural gas extraction in Pennsylvania. Environ. Res. Lett. 8:014017; doi:10.1088/1748-9326/8/1/014017. 18. Macey GP, Breech R, Chernaik M, Cox C, Larson D, Thomas D, et al. 2014. Air concentrations of volatile compounds near oil and gas production: a community-based exploratory study. Environmental Health 13:82; doi:10.1186/1476-069X-13-82. 19. McKenzie LM, Witter RZ, Newman LS, Adgate JL. 2012. Human health risk assessment of air emissions from development of unconventional natural gas resources. Sci. Total Environ. 424:79–87; doi:10.1016/j.scitotenv.2012.02.018. 20. McLeod JD, Brinkman GL, Milford JB. 2014. Emissions Implications of Future Natural Gas Production and Use in the Rocky Mountain Region. Environ. Sci. Technol. 48:13036–13044; doi:10.1021/es5029537. 21. Olaguer EP. 2012. The potential near-source ozone impacts of upstream oil and gas industry emissions. J Air Waste Manag Assoc 62: 966–977. 22. Oltmans S, Schnell R, Johnson B, Pétron G, Mefford T, Neely R. 2014. Anatomy of wintertime ozone associated with oil and natural gas extraction activity in Wyoming and Utah. Elementa: Science of the Anthropocene 2:000024; doi:10.12952/journal.elementa.000024. 23. Pacsi AP, Alhajeri NS, Zavala-Araiza D, Webster MD, Allen DT. 2013. Regional air quality impacts of increased natural gas production and use in Texas. Environ. Sci. Technol. 47:3521–3527; doi:10.1021/es3044714. 24. Pacsi AP, Kimura Y, McGaughey G, Mcdonald-Buller EC, Allen DT. 2015. Regional ozone impacts of increased natural gas use in the Texas power sector and development in the Eagle Ford shale. Environ. Sci. Technol.; doi:10.1021/es5055012. 25. Paulik LB, Donald CE, Smith BW, Tidwell LG, Hobbie KA, Kincl L, et al. 2015. Impact of natural gas extraction on PAH levels in ambient air. Environ. Sci. Technol.; doi:10.1021/es506095e. 26. Pétron G, Frost G, Miller BR, Hirsch AI, Montzka SA, Karion A, et al. 2012. Hydrocarbon emissions characterization in the Colorado Front Range: A pilot study. J. Geophys. Res. 117:D04304; doi:10.1029/2011JD016360. 27. Pétron G, Karion A, Sweeney C, Miller BR, Montzka SA, Frost G, et al. 2014. A new look at methane and non-methane hydrocarbon emissions from oil and natural gas operations in the Colorado Denver-Julesburg Basin. J. Geophys. Res. Atmos. 2013JD021272; doi:10.1002/2013JD021272. 28. Rappenglück B, Ackermann L, Alvarez S, Golovko J, Buhr M, Field RA, et al. 2014. Strong wintertime ozone events in the Upper Green River basin, Wyoming. Atmos. Chem. Phys. 14:4909–4934; doi:10.5194/acp-14-4909-2014. 29. Rich A, Grover JP, Sattler ML. 2014. An exploratory study of air emissions associated with shale gas development and production in the Barnett Shale. Journal of the Air & Waste Management Association 64:61–72; doi:10.1080/10962247.2013.832713. 30. Rodriguez MA, Barna MG, Moore T. 2009. Regional impacts of oil and gas development on ozone formation in the western United States. J Air Waste Manag Assoc 59: 1111–1118. 31. Roy AA, Adams PJ, Robinson AL. 2013. Air pollutant emissions from the development, production, and processing of Marcellus Shale natural gas. Journal of the Air & Waste Management Association 64:19–37; doi:10.1080/10962247.2013.826151. 32. Schnell RC, Oltmans SJ, Neely RR, Endres MS, Molenar JV, White AB. 2009. Rapid photochemical production of ozone at high concentrations in a rural site during winter. Nature Geosci 2:120–122; doi:10.1038/ngeo415. 33. Swarthout RF, Russo RS, Zhou Y, Hart AH, Sive BC. 2013. Volatile organic compound distributions during the NACHTT campaign at the Boulder Atmospheric Observatory: Influence of urban and natural gas sources. J. Geophys. Res. Atmos. 118:10,614–10,637; doi:10.1002/jgrd.50722. 34. Swarthout RF, Russo RS, Zhou Y, Miller BM, Mitchell B, Horsman E, et al. 2015. Impact of Marcellus Shale Natural Gas Development in Southwest Pennsylvania on Volatile Organic Compound Emissions and Regional Air Quality. Environ. Sci. Technol. 49:3175–3184; doi:10.1021/es504315f. 35. Thompson CR, Hueber J, Helmig D. 2014. Influence of oil and gas emissions on ambient atmospheric non-methane hydrocarbons in residential areas of Northeastern Colorado. Elementa: Science of the Anthropocene 2:000035; doi:10.12952/journal.elementa.000035. 36. Vinciguerra T, Yao S, Dadzie J, Chittams A, Deskins T, Ehrman S, et al. 2015. Regional air quality impacts of hydraulic fracturing and shale natural gas activity: Evidence from ambient VOC observations. Atmospheric Environment 110:144–150; doi:10.1016/j.atmosenv.2015.03.056. 37. Walters K, Jacobson J, Kroening Z, Pierce C. 2015. PM2.5 Airborne Particulates Near Frac Sand Operations. J. Environ. Health 78: 8–12. 38. Warneke C, Geiger F, Edwards PM, Dube W, Pétron G, Kofler J, et al. 2014. Volatile organic compound emissions from the oil and natural gas industry in the Uinta Basin, Utah: point sources compared to ambient air composition. Atmos. Chem. Phys. Discuss. 14:11895–11927; doi:10.5194/acpd-14-11895-2014. 39. Yuan B, Kaser L, Karl T, Graus M, Peischl J, Campos TL, et al. 2015. Airborne flux measurements of methane and volatile organic compounds over the Haynesville and Marcellus shale gas production regions. J. Geophys. Res. Atmos. 120:2015JD023242; doi:10.1002/2015JD023242. 40. Zavala-Araiza D, Sullivan DW, Allen DT. 2014. Atmospheric Hydrocarbon Emissions and Concentrations in the Barnett Shale Natural Gas Production Region. Environ. Sci. Technol. 48:5314–5321; doi:10.1021/es405770h. |
| **Category B: Findings that indicate no significantly elevated air pollutant emissions and/or atmospheric concentrations (6)** |
| 1. Bunch AG, Perry CS, Abraham L, Wikoff DS, Tachovsky JA, Hixon JG, et al. 2014. Evaluation of impact of shale gas operations in the Barnett Shale region on volatile organic compounds in air and potential human health risks. Science of The Total Environment 468–469:832–842; doi:10.1016/j.scitotenv.2013.08.080. 2. Ethridge S, Bredfeldt T, Sheedy K, Shirley S, Lopez G, Honeycutt M. 2015. The Barnett Shale: From problem formulation to risk management. Journal of Unconventional Oil and Gas Resources; doi:10.1016/j.juogr.2015.06.001. 3. Goetz JD, Floerchinger C, Fortner EC, Wormhoudt J, Massoli P, Knighton WB, et al. 2015. Atmospheric Emission Characterization of Marcellus Shale Natural Gas Development Sites. Environ. Sci. Technol.; doi:10.1021/acs.est.5b00452. 4. Rutter AP, Griffin RJ, Cevik BK, Shakya KM, Gong L, Kim S, et al. 2015. Sources of air pollution in a region of oil and gas exploration downwind of a large city. Atmospheric Environment 120:89–99; doi:10.1016/j.atmosenv.2015.08.073. 5. Song W, Chang Y, Liu X, Li K, Gong Y, He G, et al. 2015. A Multiyear Assessment of Air Quality Benefits from China’s Emerging Shale Gas Revolution: Urumqi as a Case Study. Environ. Sci. Technol. 49:2066–2072; doi:10.1021/es5050024. 6. Zielinska B, Campbell D, Samburova V. 2014. Impact of emissions from natural gas production facilities on ambient air quality in the Barnett Shale area: a pilot study. J Air Waste Manag Assoc 64: 1369–1383. |
| **Excluded (15)** |
| 1. Allen DT. 2014. Atmospheric Emissions and Air Quality Impacts from Natural Gas Production and Use. Annu Rev Chem Biomol Eng; doi:10.1146/annurev-chembioeng-060713-035938. 2. Bolden AL, Kwiatkowski CF, Colborn T. 2015. New Look at BTEX: Are Ambient Levels a Problem? Environ. Sci. Technol. 49:5261–5276; doi:10.1021/es505316f. 3. Carlton AG, Little E, Moeller M, Odoyo S, Shepson PB. 2014. The Data Gap: Can a Lack of Monitors Obscure Loss of Clean Air Act Benefits in Fracking Areas? Environ. Sci. Technol. 48:893–894; doi:10.1021/es405672t. 4. Field RA, Soltis J, Murphy S. 2014. Air quality concerns of unconventional oil and natural gas production. Environ. Sci.: Processes Impacts; doi:10.1039/C4EM00081A. 5. Lee L, Wooldridge PJ, deGouw J, Brown SS, Bates TS, Quinn PK, et al. 2015. Particulate organic nitrates observed in an oil and natural gas production region during wintertime. Atmos. Chem. Phys. 15:9313–9325; doi:10.5194/acp-15-9313-2015. 6. Levi MA. 2012. Comment on “Hydrocarbon emissions characterization in the Colorado Front Range: A pilot study” by Gabrielle Pétron et al. J. Geophys. Res. 117:D21203; doi:10.1029/2012JD017686. 7. McCawley M. 2015. Air Contaminants Associated with Potential Respiratory Effects from Unconventional Resource Development Activities. Semin Respir Crit Care Med 36:379–387; doi:10.1055/s-0035-1549453. 8. Moore CW, Zielinska B, Pétron G, Jackson RB. 2014. Air Impacts of Increased Natural Gas Acquisition, Processing, and Use: A Critical Review. Environ. Sci. Technol. 48:8349–8359; doi:10.1021/es4053472. 9. Olaguer EP, Erickson M, Wijesinghe A, Neish B, Williams J, Colvin J. 2015. Updated Methods for Assessing the Impacts of Nearby Gas Drilling and Production on Neighborhood Air Quality and Human Health. J Air Waste Manag Assoc; doi:10.1080/10962247.2015.1083914. 10. Oswald W, Harper K, Barickman P, Delaney C. 2015. Using growth and decline factors to project VOC emissions from oil and gas production. J Air Waste Manag Assoc 65:64–73; doi:10.1080/10962247.2014.960104. 11. Pekney NJ, Veloski G, Reeder M, Tamilia J, Rupp E, Wetzel A. 2014. Measurement of atmospheric pollutants associated with oil and natural gas exploration and production activity in Pennsylvania’s Allegheny National Forest. Journal of the Air & Waste Management Association 64:1062–1072; doi:10.1080/10962247.2014.897270. 12. Pétron G, Frost GJ, Trainer MK, Miller BR, Dlugokencky EJ, Tans P. 2013. Reply to comment on “Hydrocarbon emissions characterization in the Colorado Front Range—A pilot study” by Michael A. Levi. J. Geophys. Res. Atmos. 118:236–242; doi:10.1029/2012JD018487. 13. Warneke C, Veres P, Murphy SM, Soltis J, Field RA, Graus MG, et al. 2015. PTR-QMS versus PTR-TOF comparison in a region with oil and natural gas extraction industry in the Uintah Basin in 2013. Atmos. Meas. Tech. 8:411–420; doi:10.5194/amt-8-411-2015. 14. Weinhold B. 2012. The Future of Fracking: New Rules Target Air Emissions for Cleaner Natural Gas Production. Environ Health Perspect 120:a272–a279; doi:10.1289/ehp.120-a272. 15. Wennberg PO, Mui W, Wunch D, Kort EA, Blake DR, Atlas EL, et al. 2012. On the Sources of Methane to the Los Angeles Atmosphere. Environ. Sci. Technol. 46:9282–9289; doi:10.1021/es301138y. |
